# Normally lethal amino acid substitutions suppress an ultramutator DNA Polymerase δ variant

**DOI:** 10.1038/srep46535

**Published:** 2017-04-18

**Authors:** Daniel G. Dennis, Jill McKay-Fleisch, Kaila Eitzen, Ian Dowsett, Scott R. Kennedy, Alan J. Herr

**Affiliations:** 1 Department of Pathology, University of Washington, Seattle, Washington 98195, USA.

## Abstract

In yeast, the *pol3*-*01,L612M* double mutant allele, which causes defects in DNA polymerase delta (Pol δ) proofreading (*pol3*-*01*) and nucleotide selectivity (*pol3*-*L612M*), confers an “ultramutator” phenotype that rapidly drives extinction of haploid and diploid MMR-proficient cells. Here, we investigate antimutator mutations that encode amino acid substitutions in Pol δ that suppress this lethal phenotype. We find that most of the antimutator mutations individually suppress the *pol3*-*01* and *pol3*-*L612M* mutator phenotypes. The locations of many of the amino acid substitutions in Pol δ resemble those of previously identified antimutator substitutions; however, two novel mutations encode substitutions (R674G and Q697R) of amino acids in the fingers domain that coordinate the incoming dNTP. These mutations are lethal without *pol3*-*L612M* and markedly change the mutation spectra produced by the *pol3*-*01,L612M* mutator allele, suggesting that they alter nucleotide selection to offset the *pol3*-*L612M* mutator phenotype. Consistent with this hypothesis, mutations and drug treatments that perturb dNTP pool levels disproportionately influence the viability of *pol3*-*L612M,R674G* and *pol3*-*L612M,Q697R* cells. Taken together, our findings suggest that mutation rate can evolve through genetic changes that alter the balance of dNTP binding and dissociation from DNA polymerases.

Cells must accurately duplicate their genome each division to ensure that their descendants inherit the proven genetic traits of past generations. The greatest contribution to high fidelity DNA synthesis comes from the inherent nucleotide selectivity of the main replicative DNA polymerases, which make one misinsertion every 10^5^ to 10^6^ base-pairs. Extension of the resulting mispaired primer termini by replicative polymerases normally occurs inefficiently^1^,^2^,^3^,^4^. Pausing provides time for mismatched primer termini to partition to an intrinsic or extrinsic 3′ → 5′ proofreading exonuclease^5^,^6^,^7^, which removes the errant 3′ nucleotide. If polymerases extend the mismatch before proofreading occurs, post-replicative mismatch repair (MMR) complexes recognize the distorted duplex DNA and initiate excision and re-synthesis of the nascent strand^8^,^9^,^10^,^11^,^12^. Together, nucleotide selectivity, proofreading, and MMR lower the DNA replication error rate to less than 1 × 10^−9^ mutations per base pair per cell division^13^.

In eukaryotes, DNA polymerases epsilon (Pol ε) and delta (Pol δ) are thought to perform the bulk of leading and lagging strand DNA synthesis, respectively^14^,^15^,^16^. Proofreading-deficient Pol ε and Pol δ variants elevate mutation rates in yeast^8^,^17^,^18^,^19^ and mouse cells^20^,^21^,^22^. Consistent with the hypothesis that high DNA replication fidelity restrains neoplasia, Pol ε and Pol δ proofreading defects increase cancer risk in mice^20^,^21^,^22^ and recently have been found in highly mutated human colorectal and endometrial cancers^23^,^24^,^25^,^26^,^27^,^28^, complementing studies from the 1990’s linking the Lynch cancer susceptibility syndrome to MMR defects^29^.

Since nucleotide selectivity, proofreading and MMR act redundantly to limit the transmission of DNA polymerase errors, combined defects in any two of these activities lead to strong synergistic mutator phenotypes in diploid yeast, with mutation rates 1000–50,000 fold more than that of wild-type cells^8^,^19^,^30^,^31^,^32^. Recent evidence suggests that such “ultramutator” cells may emerge spontaneously in certain human cancers. The most dramatic demonstration comes from patients with bi-allelic MMR deficiencies who spontaneously developed brain tumors with additional mutations affecting nucleotide selectivity or proofreading^33^.

The emergence of cell lineages with synergistic mutator alleles conforms to evolutionary theory. Mutator alleles hitch-hike on the fitness benefits of the adaptive mutations they generate^34^,^35^,^36^,^37^,^38^, and an additional increase in mutation rate fuels even more rapid adaptation. But the accumulation of deleterious mutations in mutator cells takes a toll. In bacteria and both haploid and diploid yeast, mutator phenotypes that exceed an error threshold drive extinction of the mutator lineage within a few cell divisions due to inactivation of essential genes, a process we refer to as *Error*-*induced Extinction (EEX*)^32^,^39^,^40^,^41^,^42^,^43^. Evidence also exists for error thresholds in multicellular organisms. Mice that are homozygous for mutant alleles that cause defects in MMR and either Pol δ or Pol ε proofreading are embryonic lethal^22^.

Intriguingly, human tumor cells with tandem deficiencies in MMR and proofreading do not appear to exceed a mutation load of ~250 × 10^−6^ mutations/bp^25^,^33^. Diploid yeast cells with mutation rates near the threshold of error-induced extinction carry similar mutation burdens of ~70 × 10^−6^ mutations/bp and exhibit highly variable colony forming capacity^32^. Thus, the apparent upper limit of mutation load in ultramutator tumors may derive from an attrition of the most highly mutated cells, whose probability of getting a lethal mutation in the next round of replication increases proportionately with the number of recessive lethal mutations they carry.

Yeast cells undergoing *EEX* experience strong selection pressure for *eex* mutants with “antimutator” alleles that lower mutation rates to tolerable levels^38^,^39^,^44^,^45^,^46^,^47^. In this present study, we investigate *eex* mutations that suppress the ultramutator phenotype of a double mutant allele (*pol3*-*01,L612M*) created by combining the classic *pol3*-*01* mutation, which compromises proofreading, with a mutation encoding an L612 to M substitution in the Pol δ active site^32^,^48^. L612 is highly conserved and forms part of the binding site for the incoming dNTP^49^. Early studies by Reha-Krantz and colleagues with Phage T4 polymerase established that the equivalent L412M substitution conferred a mutator phenotype^50^. The corresponding substitution in yeast Pol δ increases mutation rate modestly, producing primarily base-substitution errors^48^,^51^. The same change in human Pol δ (L606M) increases the error rate *in vitro*^52^ and was recently observed in a hyper-mutated brain tumor from one of the above patients with bi-allelic MMR deficiency^33^. We previously showed that the *pol3*-*01,L612M* allele induces both haploid and diploid yeast cells to undergo error-induced extinction^32^, with the inferred mutation rate of *pol3*-*01,L612M* diploids being ~50,000 times greater than that of wild-type cells^32^. We found that the *eex* mutations that suppressed *pol3*-*01,L612M* lethality exhibited a wide range of antimutator effects (reprinted in Table 1)^32^. In theory, the *eex* alleles could specifically suppress the *pol3*-*01* or *pol3*-*L612M* mutator alleles or exert a general antimutator effect on Pol δ fidelity. Here, we explore this question of allele-specificity and examine the structural implications of the *eex* substitutions in order to understand their underlying antimutator mechanisms.

## Results

### Interactions between mutator and antimutator alleles

**Figure 1:**
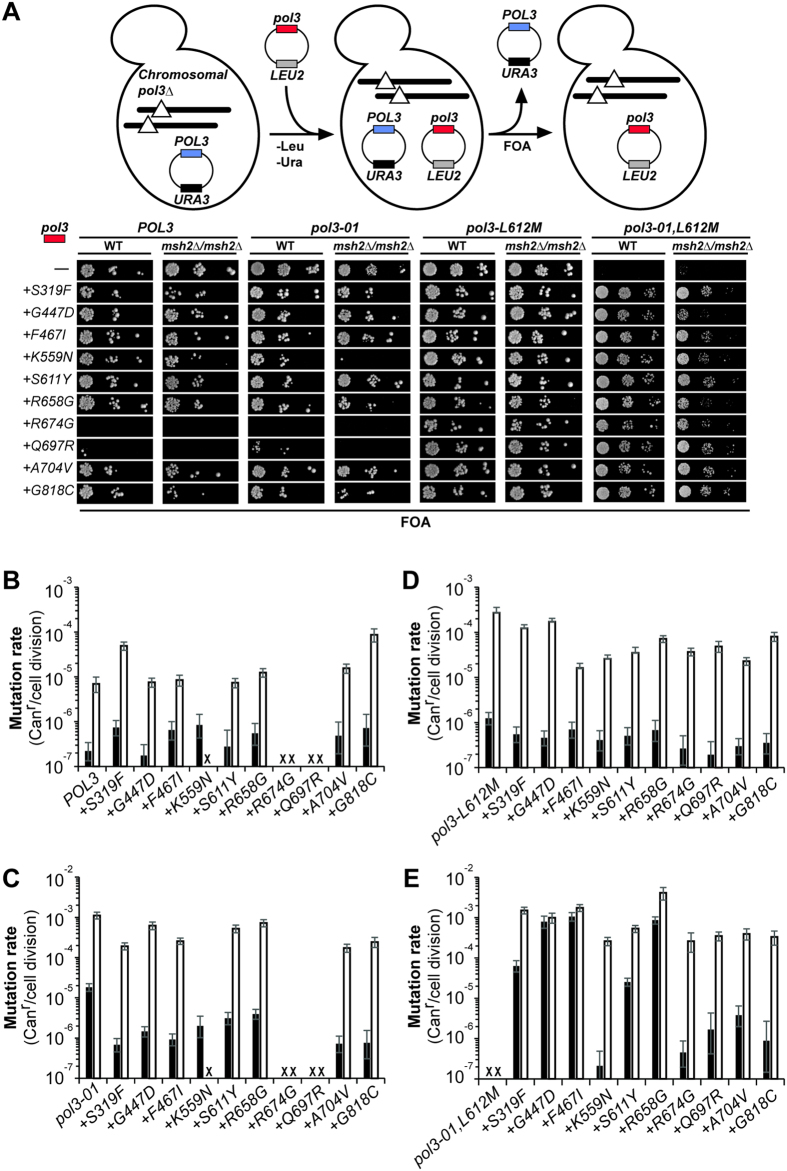
Viability and mutation rates conferred by *eex* alleles. (**A**) *Top*. Plasmid shuffling scheme. Diploid plasmid shuffling strains with chromosomal deletions of *POL3* (lines with triangles) complemented by a *POL3–URA3* plasmid (blue) were transformed with *LEU2* plasmids (red) expressing various *POL3* alleles (*pol3*-*LEU2*) on plates lacking leucine and uracil (-Leu -Ura). *Bottom*. The above *pol3*-*LEU2* transformants were then plated on FOA media in 10-fold serial dilutions to assess viability. FOA-resistant colonies were used as replica cultures in a fluctuation assay to determine mutation rates conferred by (**B**) *pol3*-*eex* alleles, (**C**) *pol3*-*01,eex*, (**D**) *pol3*-*L612M,eex* and (**E**) *pol3*-*01,L612M,eex* (reprinted from Herr *et al*.^32^). Black bars indicate MMR proficient cells (BP8001). White bars indicate *msh2*Δ/*msh2*Δ cells (BP9101). “X” indicates no growth.

To test the allele-specificity of the antimutator alleles, we generated *pol3*-*01,eex* and *pol3*-*L612M,eex* double mutant alleles, as well as *pol3*-*eex* single mutant alleles, and then examined their influence on viability and mutation rates in the presence and absence of MMR. Mutations conferring MMR-deficiency are synthetically lethal with *pol3*-*01* in haploids, but not in diploids. Thus, we utilized wild-type and Msh2-deficient diploid “plasmid shuffling” strains, which were engineered to be hemizygous for *CAN1* to permit measurement of forward mutation rates^32^. The strains carry deletions of both chromosomal *POL3* genes, complemented by a *POL3*-*URA3*-*CEN* plasmid. We transformed these cells with wild-type and mutant *POL3* alleles carried on *LEU2*-*CEN* plasmids, and then assessed their influence on viability by plating each strain-plasmid combination on FOA plates, which selects for loss of the *POL3*-*URA3* plasmid (~1 in 100 cells) (Fig. 1A). For viable strains, the FOA-resistant colonies were then used as replica colonies for mutation rate determination by fluctuation analysis^53^,^54^.

MMR-proficient diploids grew with most of the mutant *eex* alleles as the sole source of Pol δ, indicating that the corresponding amino acid substitutions do not substantially impair Pol δ activity (Fig. 1A). The exceptions were the *pol3*-*eex* and *pol3*-*01,eex* alleles with *R674G* or *Q697R*. The lethality of these alleles suggests that the R674G and Q697R substitutions inhibit Pol δ function in the absence of L612M. Three of the *pol3*-*eex* alleles (*S319F, F467I*, and *K559N*) increased mutation rate significantly in the presence of MMR by a likelihood ratio test, recently developed by Zheng^54^ (Fig. 1B, Table 1). In the absence of Msh2, four alleles (*pol3*-*S319F, pol3*-*R658G, pol3*-*A704V*, and *pol3*-*G818C*) increased mutation rates significantly, suggesting that the corresponding polymerases create errors that MMR corrects. In contrast, the absence of a synergistic effect between *pol3*-*F467I* and *msh2Δ*, suggests that the errors triggered by Pol δ-F467I are not subject to MMR. Unexpectedly, the *pol3*-*K559N* allele conferred poor colony forming capacity in the absence of MMR (Fig. 1A), which precluded measuring mutation rates. Proofreading deficiency may exacerbate this synthetic phenotype, as the *pol3*-*01,K559N* allele was synthetically lethal with *msh2*Δ (Fig. 1A).

Although some of the *eex* alleles confer mutator phenotypes on their own, they exert clear antimutator effects on *pol3*-*01* and *pol3*-*L612M* (Fig. 1C,D). All non-lethal *eex* alleles suppressed the *pol3*-*01* mutator phenotype to levels observed with previously isolated *pol3*-*01,eex* alleles^39^. The *eex* alleles exerted more modest antimutator effects on *pol3*-*L612M* (Table 1), which were more apparent in *msh2Δ* cells, where the majority lowered mutation rates more than 3-fold (Table 1). Thus, although the *eex* alleles could theoretically have been specific to *pol3*-*01* or *pol3*-*L612M*, most appear to increase fidelity by perturbing an aspect of Pol δ activity required for both mutator mechanisms.

### Influence of antimutator alleles on *pol3*-*01,L612M* mutation spectra

In order to understand how the *eex* mutations altered the *pol3*-*01,L612M* mutator phenotype, we performed whole genome sequencing of four Msh2-deficient *pol3*-*01,L612M,eex* strains that express Pol δ variants with amino acid substitutions near the polymerase active site (S611Y, R674G, Q697R, and K559N). The canavanine-resistance mutation rate exhibited by each of these strains is at least 40 times higher than the *POL3* Msh2-deficient control (Fig. 1E, Table 1); thus, most mutations in these strains should derive from errors caused by the mutant Pol δ variants. We obtained independent isolates of these strains by plasmid shuffling. After they formed a colony of 10^5^ to 10^6^ cells, which corresponds to 17–20 generations of growth, we isolated single cells from the original colonies and allowed them to form new colonies. We then identified the accumulated mutations by whole genome sequencing, scoring both the frequency of different classes of mutations as well as their relative fraction of the total mutations observed. As controls, we sequenced similarly isolated MMR-deficient strains expressing *pol3*-*01, pol3*-*L612M*, or both *POL3* and *pol3*-*01,L612M (POL3/pol3*-*01,L612M*). We relied on these “episomally heterozygous” *POL3/pol3*-*01,L612M* cells to define the spectrum of mutations generated by Pol δ-01,L612M, since cells expressing only the *pol3*-*01,L612M* allele are inviable.

The MMR-deficient *POL3/pol3*-*01,L612M* isolates averaged more than 1700 mutations per genome (Table S1). If we assume that these mutations occurred over ~20 generations, the Pol δ-01,L612M polymerase caused a genome-wide mutation rate of 89 mutations per cell division (Table S1). The *pol3*-*01* strains also averaged more than 1700 mutations per genome, while *pol3*-*L612M* strains averaged only 566 mutations per genome, consistent with a lower mutation rate (Table S1). Thus, although heterozygous, the *pol3*-*01,L612M* allele generates sufficient numbers of mutations for meaningful comparisons with the mutation spectra from other strains.

**Figure 2:**
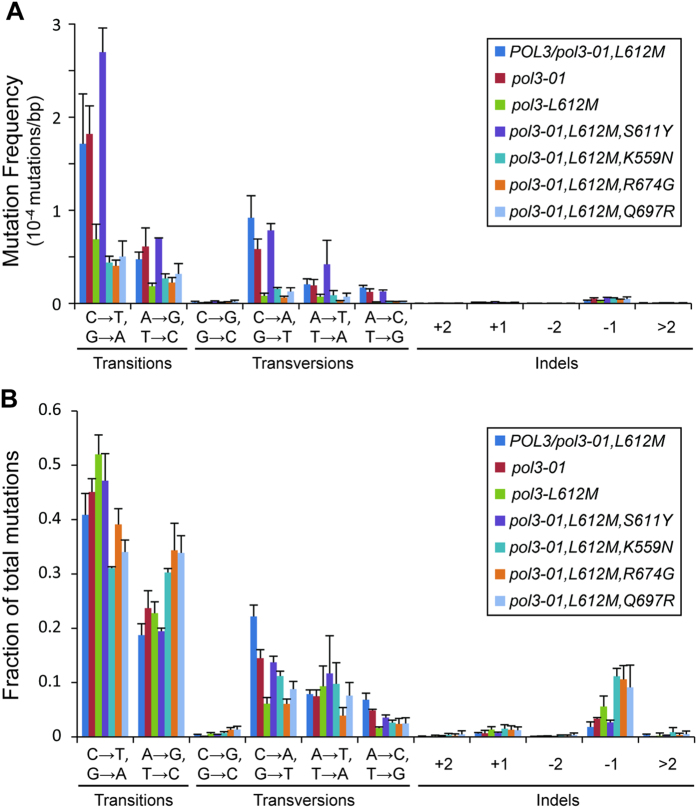
Mutation Spectra of *pol3*-*01,L612M,eex* cells. We clonally isolated cells expressing mutator alleles after 17–20 generations of growth and performed whole genome sequencing. (**A**) Mutation frequencies. The frequencies of transitions and transversions were determined by dividing the number of observations of each class by the number of scored sites in the genome that could give rise to that mutation. The frequency of each class of insertion/deletion mutation (indels) were determined by dividing the number of observations by the total number of scored sites. (**B**) Mutation Fractions. The fraction of each class of mutation was determined by dividing the number of observation of each class by the total number of observed mutations. The error bars for both (**A**) and (**B**) represent the standard deviations of the mutation frequencies and fractions obtained from independent isolates of the same genotype.

As observed previously in a *POL3/pol3*-*01,L612M* MMR-proficient diploid^32^, the most abundant mutations observed in the *POL3/pol3*-*01,L612M* MMR-deficient cells were C → T/G → A transitions, followed by A → G/T → C transitions and C → A/G → T transversions, which were 50% less frequent than C → T/G → A transitions (Fig. 2, Table S1). Like *POL3/pol3*-*01,L612M* cells, cells with *pol3*-*01* or *pol3*-*L612M* generated roughly half as many A → G/T → C transitions as C → T/G → A transitions; however, both had a lower fraction of C → A/G → T transversions than the *POL3/pol3*-*01,L612M* cells (Fig. 2B). The MMR-deficient *pol3*-*01,L612M,S611Y* cells, which averaged more than 2400 mutations per genome, exhibited a mutation spectrum similar to *pol3*-*01* cells (Fig. 2B). Cells expressing *pol3*-*01,L612M,eex* alleles with the *K559N, R674G*, or *Q697R* mutations had overall mutation frequencies that were similar to *pol3*-*L612M* cells (Fig. 2A). Like *pol3*-*L612M* cells, they produced a smaller fraction of C → A/G → T transversion mutations than *POL3/pol3*-*01,L612M* cells. However, each *pol3*-*01,L612M,eex* strain produced proportionally similar levels of C → T/G → A and A → G/T → C transitions, in contrast to the *POL3/pol3*-*01,L612M, pol3*-*01*, and *pol3*-*L612M* strains (Fig. 2B, Table S1). The striking similarity in mutation spectra induced by these *eex* alleles suggests that they suppress the ultramutator phenotype of *pol3*-*01,L612M* by a common mechanism that influences nucleotide selectivity.

### Mutations and drug treatments known to perturb dNTP pool levels influence the viability of cells expressing *pol3*-*L612M,R674G* or *pol3*-*L612M,Q697R*

**Figure 3:**
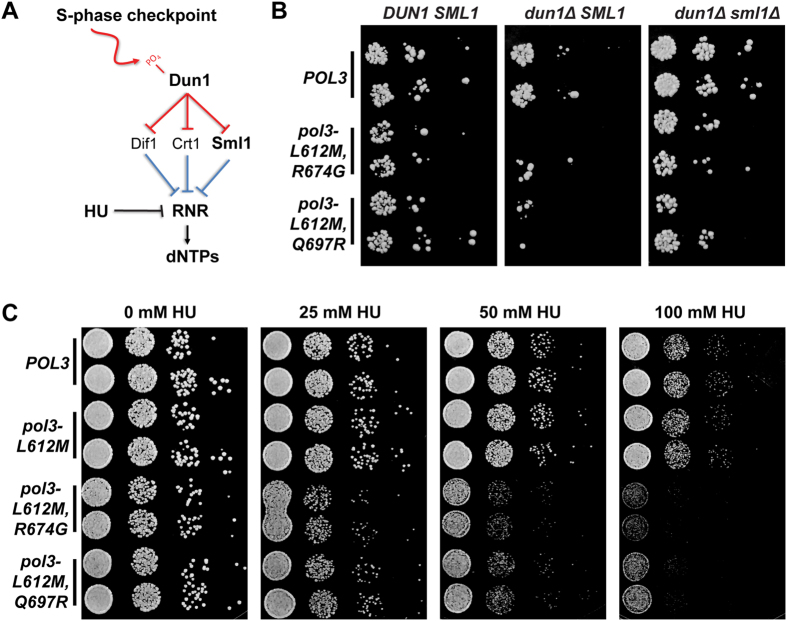
Sensitivity of *pol3*-*L612M,R674G* and *pol3*-*L612M,Q697R* strains to conditions that modify dNTP pool levels. (**A**) Regulation of dNTP synthesis. Phosphorylated Dun1 increases dNTP synthesis by negatively regulating Dif1 (Damage-regulated Import facilitator 1)^78^ Crt1, (Constitutive RNR Transcription 1)^79^, and Sml1. These three proteins repress either RNR gene expression or RNR assembly. (**B**) Genetic interactions with *dun1*Δ and *sml1*Δ. Two independent plasmid shuffling strains per genotype were plated onto FOA media in ten-fold serial dilutions. (**C**) Sensitivity to HU. Two independently shuffled strains per genotype were plated in ten-fold serial dilutions onto media containing increasing concentrations of HU.

Nucleotide selectivity is influenced by the composition and levels of dNTP pools, which are regulated in yeast by the Damage uninducible (Dun) 1 kinase as part of the S-phase checkpoint^55^ and during normal dNTP homeostasis^56^,^57^. Elimination of Dun1 (*dun1*Δ) suppresses the lethality of the *pol3*-*01,L612M* ultramutator allele and the mutator phenotypes of most *pol3*-*01,L612M,eex* alleles^32^, as well as *pol3*-*01*^58^, *pol2*-*4*^57^, and *pol3*-*R696W*^59^. Intriguingly, *pol3*-*01,L612M,R674G* and *pol3*-*01,L612M,Q697R* are synthetically lethal with *dun1*Δ^32^, suggesting that the R674G and Q697R substitutions may lower dNTP binding affinity of Pol δ, thereby imposing a requirement for higher dNTP pools. Dun1 increases dNTP pools by upregulating ribonucleotide reductase (RNR) — the rate limiting enzyme for dNTP synthesis — in part, by targeting Suppressor of *mec1* lethality (Sml)1^60^, which interferes with RNR complex assembly (Fig. 3A)^61^. Previous work has shown that while *dun1*Δ mutant cells have a two-fold reduction in dNTP pools relative to wild-type cells, *dun1*Δ *sml1*Δ mutant cells have a 2-fold increase in dNTP pools relative to wild-type cells^62^.

To test the hypothesis that insufficient dNTP pools drives the synthetic lethality between *dun1*Δ and the *R674G* or *Q697R* mutations, we assessed the viability of *dun1*Δ and *dun1*Δ *sml1*Δ haploid strains expressing the *pol3*-*L612M,R674G* or *pol3*-*L612M,Q697R* alleles. While Dun1 deficiency impaired growth of cells expressing either allele, Sml1 deficiency ameliorated this effect (Fig. 3B). As a second test of the hypothesis, we assessed the sensitivity of *DUN1*-proficient *pol3*-*L612M,R674G* or *pol3*-*L612M,Q697R* mutants to increasing concentrations of hydroxyurea (HU), which blocks dNTP synthesis by inhibiting RNR activity (Fig. 3A). We found that both *pol3*-*L612M,eex* alleles impaired colony growth at concentrations of HU that were tolerated by cells expressing *POL3* or *pol3*-*L612M* (Fig. 3C). These observations support the hypothesis that R674G and Q697R enhance replication fidelity by reducing the dNTP binding affinity of Pol δ.

## Discussion

The evolution of mutation rate occurs through the acquisition of mutator and antimutator traits that optimize DNA replication fidelity^63^. The most radical antimutator innovations of evolution are unquestionably proofreading and MMR, which involved the recruitment of protein domains or entire complexes to enhance the accuracy of DNA replication. But most mutator and antimutator alleles likely encode single amino acid substitutions in proteins already involved in DNA replication. The mechanism by which a mutator allele elevates mutation rate may determine what antimutator mutations restore fidelity. Both general and allele-specific antimutators may be possible. We analyzed antimutator alleles that suppress a synergistic mutator phenotype caused by two different mutator alleles affecting Pol δ nucleotide selectivity and proofreading. We found that most antimutator mutations exert a suppressive effect on each mutator allele individually, suggesting that they limit an aspect of Pol δ activity required for all mutagenesis, such as the extension of mispaired primer termini. However, we did find allele-specific interactions between *pol3*-*L612M* and the *R674G* and *Q697R* mutations, which influenced cellular viability. In what follows, we discuss the basis for the ultramutator phenotype of *pol3*-*01,L612M* and how the amino acid substitutions encoded by the antimutator mutations may influence replication fidelity and allele-specific viability in light of their locations within the Pol δ structure.

Kinetic experiments in the late 1980’s revealed that high-fidelity DNA polymerases extend mispaired primer termini inefficiently, which augments proofreading^1^,^2^,^3^,^4^,^64^. In the absence of proofreading, Pol δ can eventually extend errors into duplex DNA^65^. Biochemical studies indicate that the L612M substitution lowers this kinetic barrier, allowing Pol δ to frequently extend mispairs even with an intact proofreading domain^66^. If failure to proofread mispaired primer termini was the only source of Pol δ-L612M infidelity, the mutator phenotype of *pol3*-*01* would be epistatic to that of *pol3*-*L612M*. Instead, the robust *pol3*-*01,L612M* mutator phenotype far exceeds that of the individual *pol3*-*01* or *pol3*-*L612M* alleles^32^. Thus, in addition to promoting mispair extension, the L612M substitution must also decrease nucleotide selectivity, which is supported by the distinctive *pol3*-*L612M* error signature^14^ and the increase in C → A/G → T transversions in the *POL3/pol3*-*01,L612M* strain. Of course, increased mispair extension and lowered nucleotide selectivity may derive from the same phenomenon, such as increased dNTP binding stability or higher catalytic activity.

**Figure 4:**
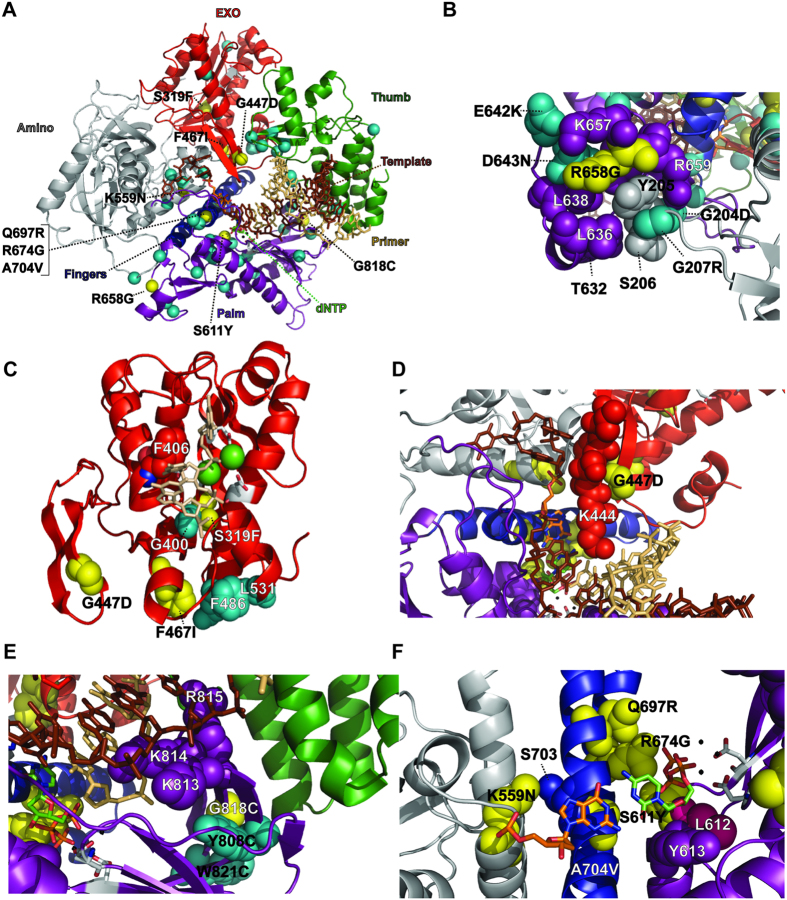
Locations of *eex* amino acid substitutions in the *S. cerevisiae* Pol δ structure. The *S. cerevisiae* Pol δ structure^49^ (Protein database accession code 3IAY) is shown as a schematic diagram of the α-carbon backbone (rendered in PyMol). Structural domains are color coded as follows: amino (gray), exo (red), palm (purple), fingers (blue) and thumb (green). Yellow spheres correspond to *eex* mutations from the present study and are labeled to indicate the amino acid substitution; light blue spheres correspond to antimutator mutations from a previous study^39^. The incoming dNTP is denoted by green CPK sticks and the template nucleotide by orange CPK sticks. The primer DNA is represented by tan sticks and the template DNA, by brown sticks. Important non-mutated residues proximal to the antimutator substitutions are shown as space-filling spheres and color coded according to the domain. Active-site carboxylate side chains are gray CPK sticks coming out of the purple (palm) or red (exo) ribbons. Metal ions are small black spheres. (**A**) Overall distribution of *eex* amino acid substitutions. The α-carbons are indicated by color coded spheres. (**B**) *eex* substitutions affecting an interaction between the amino and palm domains. (**C**) *eex* substitutions with structural roles in exonuclease domain. The last three nucleotides of the DNA primer strand from the RB69 Pol Editing structure^6^ (Protein database accession code 1CLQ) were placed in the Pol δ exo active site (tan sticks) by aligning the conserved exo domain motifs of RB69 Pol and Pol δ in Pymol. (**D**) Interaction of the β-hairpin of the exonuclease domain with the primer strand. (**E**) G818C and the KKR motif implicated in binding of duplex DNA in the minor groove^7^,^49^. (**F**) Amino acid substitutions affecting the polymerase active site. The ribose of the incoming dNTP stacks on Y613 and L612 (pink spheres). The γ-phosphate of the incoming dNTP contacts R674 and the primary amino group of S611. Q697 forms a hydrogen bond with R674, while K559N forms a hydrogen bond with S703.

Pol δ resembles a right hand with five domains including the amino-terminal domain (amino), the exonuclease domain (exo), the palm (containing the catalytic metal ions for DNA synthesis), the fingers, and the thumb (Fig. 4A)^49^. Many of the antimutator substitutions are relatively far from the polymerase active site, near previously identified substitutions encoded by mutations that suppress the *pol3*-*01* mutator phenotype^39^. The R658G substitution alters an interaction between the palm and the amino domains, also affected by the antimutator substitutions G204D, G207R, E642K, and D643N (Fig. 4B)^39^,^67^. S319F resides in the central β-sheet located in the heart of the exo domain, adjacent to the G400S antimutator substitution (Fig. 4C)^39^. The F467I substitution affects a helix-loop-helix motif in the exo domain that interacts with the backbone of the primer strand and contains the L531P and F486S antimutator substitutions (Fig. 4C)^39^. The third substitution in the exo domain, G447D, alters a β-hairpin that contacts the DNA during polymerization (Fig. 4C,D)^68^,^69^. Consistent with a role in DNA binding stability, Reha-Krantz and colleagues previously found that a different substitution at this position, G447S, suppresses frameshift mutations, while G447D increases the rate of -1 frameshift mutations^70^. Finally, the G818C substitution maps to the palm domain in a different β-hairpin affected by the Y808C and W821C antimutator substitutions. This β-hairpin contains the highly conserved KKR motif (Figure S1), which binds the minor groove of the primer•template at the −3 to −5 positions and may allow Pol δ to sense deviations from Watson-Crick base-pairing geometry in the newly synthesized DNA and initiate proofreading even after mispair extension^7^,^49^ (Fig. 4E).

The remaining antimutator substitutions from this study alter the polymerase active site, which is formed by elements of the amino, palm and fingers domains (Fig. 4F). The fingers domain is a dynamic structure that rapidly moves from an open to closed conformation upon dNTP binding. Closure of the fingers stabilizes correct dNTP•template interactions and may help the polymerase to reject incorrect nucleotides^47^. The A704V substitution occurs at the interface between the two fingers, adjacent to critical residues (N705-Y708) that form the binding site for the template•dNTP basepair. K559N resides in part of the amino domain previously implicated in *pol3*-*01* suppression (Figure S1)^39^,^47^, and forms a hydrogen bond to S703 of the fingers. The remaining substitutions (S611Y, R674G, and Q697R) alter highly conserved residues involved in dNTP binding. R674 in the fingers domain directly interacts with the γ-phosphate of the incoming dNTP through its guanido group^49^. Q697, also in the fingers domain, positions R674 to contact the incoming dNTP, while S611 in the palm domain interacts with the dNTP γ-phosphate and the guanido group of R674 (Fig. 4F).

As we proposed previously^47^, antimutator substitutions that impair DNA binding, either during polymerization or during proofreading, may promote access of mispaired primer termini to other DNA repair pathways that remove the error. Conceivably, some structural perturbations may propagate to the polymerase active site in a manner that enhances nucleotide selectivity. A more compelling case for enhanced nucleotide selectivity can be made for those substitutions that directly impact the polymerase active site and alter the mutation spectrum of *pol3*-*01,L612M* mutator cells. The most dramatic of these, R674G, eliminates a key interaction between the fingers and the γ-phosphate of the incoming dNTP. The Q697R substitution may closely phenocopy this dNTP binding defect by displacing the R674 sidechain through charge repulsion between the two arginine side chains. The hypothesis that R674G and Q697R weaken Pol δ dNTP binding affinity is supported by the lethal phenotypes of the *pol3*-*R674G* and *pol3*-*Q697R* alleles, the suppression of this lethality by *L612M*, and the sensitivity of *pol3*-*L612M,R674G* and *pol3*-*L612M,Q697R* strains to mutations and drug treatments known to lower dNTP pool levels. Defects to dNTP binding will necessarily also impact the efficiency of mispair extension. Depending on the genetic background and growth conditions, a primer extension defect may promote the correction of Pol δ errors by alternative editing pathways (in the presence of *DUN1* and *L612M*) or potentially induce lethal replication stress (in the absence of *DUN1* or *L612M* or the presence of HU). Exploring the above models will require detailed biochemical studies of the mutant polymerases as well as genetic screens for genes encoding candidate alternative editing activities.

Ultramutator phenotypes are inherently unstable in yeast. It remains to be shown whether this is also true in cancer cells. The apparent mutation threshold observed in tumors deficient in proofreading and MMR^33^ may simply reflect the most probable mutation load of an ultramutator cancer cell at the time of malignant transformation. Importantly, the sequencing methodologies used in these studies are blind to whether individual cells within the ultramutated tumors continue to fix mutations at a high rate. Nevertheless, the existence of a mutation threshold in diploid yeast supports the hypothesis of an error threshold in human cancers. If a threshold exists, the outer limits of mutation load may be determined by a loss of replicative fitness or by the suppressive effects of immune recognition of newly arising neoantigens. Given enough cell proliferation, either mechanism may favor the emergence of antimutator subpopulations of cells within the tumor. If antimutators do arise, the underlying mechanism may create new vulnerabilities that could be exploited for therapy. For instance, antimutators that suppress the mutator phenotype by promoting dissociation of the polymerase from mispaired primer•templates may be dependent on a “targetable” DNA repair pathway to correct the errors. Likewise, antimutators that destabilize dNTP binding in the active site may confer sensitivity to drugs that dampen dNTP pool levels.

## Materials and Methods

### Media and Growth Conditions

Yeast were grown at 30 °C using YPD, synthetic complete (SC) media or SC ‘drop-out’ media deficient in defined amino acids to select for prototrophic genetic markers^71^. Premade nutrient supplements for SC and SC lacking uracil and leucine were purchased from Bufferad. Other drop-out nutrient supplements were made as described^71^ from individual components purchased from Sigma-Aldrich or Fisher Scientific. To select for Ura^−^ cells during plasmid shuffling, we used SC media containing 1 mg/ml 5-fluroorotic-acid (FOA; Zymo Research)^72^. For mutation rate assays, we used SC plates containing 60 μg/ml canavanine and 200 μg/L geneticin (G418, Sigma-Aldrich) with mono-sodium-glutamate (MSG) (1 g/L) as the nitrogen source rather than ammonium sulfate^73^. To select for hygromycin resistance during strain construction (see below), we used YPD plates with 300 μg/ml hygromycin (Corning). For hydroxyurea (HU; MP Biomedicals, LLC) plates, we dissolved HU in 2xYPD at concentrations of 200 mM, 100 mM, 50 mM, and 0 mM. We filter-sterilized each solution, and then combined it with sterile 2x (4%) agar to obtain plates with 100 mM, 50 mM, 25 mM, and 0 mM HU.

### Yeast Plasmids and Strains

#### Plasmids

pGL310^17^,^74^ is derived from YCp50 (*CEN4/ARS1/URA3*)^75^ and carries *POL3* under control of the endogenous promoter. YCplac111*POL3* and YCplac111*pol3* derivatives^39^ are derived from YCplac111 (*CEN6/ARS1/LEU2*)^76^, and carry the wild-type or mutant *pol3* coding and regulatory sequences, cloned between the HindIII and EcoRI restriction sites. Construction of YCplac111*pol3*-*L612M* and YCplac111*pol3*-*01,L612M* were described previously^48^. We utilized conventional cloning or the Quikchange protocol to re-engineer the *eex* mutations into YCplac111*POL3* and YCplac111*pol3* vectors.

#### Strains

The MMR proficient (BP8001) and MMR deficient (BP9101) diploid *POL3* plasmid shuffling strains as well as the Dun1-proficient (BP7801) and *dun1*Δ (BP8101) haploid *POL3* plasmid shuffling strains have been described previously^32^. To create a *dun1*Δ *sml1*Δ strain derivative of BP8101 (BP8101-T1), we deleted the *SML1* gene by transforming the strain with the hygromycin-resistance gene (*HphMX4*), amplified from pFvL100^77^ using the previously described sml1U and sml1D primers^57^, which contain 50 nucleotides upstream and downstream of the *SML1* coding sequence. We confirmed the deletion/insertion using flanking primers sml1-744F (5′-CGCGCTGAGCCCAAACGGGCTCCACTA-3′) and sml1-1745R (5′-GGCTCCTGTGTGACTCTATGGGAGGGAAGGA-3′), which amplify a 960 bp fragment in *SML1* cells and a 2414 bp fragment in the *sml1*Δ::*HphMX* cells.

### Plasmid shuffling

Plasmid shuffling with pGL310-containing strains has been described previously^17^,^39^,^72^. Cells transformed with YCplac111*pol3* plasmids, YCplac111*POL3* (positive control), or YCplac111 (empty vector control) were plated on SC lacking uracil and leucine. After two to three days incubation at 30 °C, individual colonies were picked and dispersed in sterile H_2_O. Serial dilutions containing approximately 10^5^, 10^4^, 10^3^, and 10^2^ cells were plated on SC or SC with FOA to select for cells that had spontaneously lost pGL310.

### Mutation Rates

Mutation rates were determined by fluctuation analysis of canavanine-resistant mutants in replica cultures^53^ as described previously^32^. To measure forward mutation rates in diploids, we engineered our *pol3Δ/pol3Δ* deletion strains to be hemizygous for the *CAN1* gene. Forward mutation rate assays using hemizygous target genes in diploid yeast are confounded by high rates of mitotic recombination, which produce phenotypically mutant colonies. Thus, these strains carry the *kanMX* transgene downstream of the functional *CAN1* allele to allow selection against mitotic recombinants on canavanine selection plates with G418. This approach lowers the background of Can^r^ colonies down to the level observed in *CAN1* haploid cells.

Mutation rates and statistical tests were calculated using the R statistical package, rSalvador^54^, from the number of mutant colonies in each replica by first estimating *m* by maximum likelihood using newton.LD.plating and then dividing by the number of cell divisions inferred from the average number of colony forming units in the replica cultures. Confidence intervals were calculated using confint.LD.plating, which relies on likelihood ratios. Statistical comparisons between mutation rates were performed using a likelihood ratio test (LRT.LD.plating). Bonferroni corrections were made in R using p.adjust.

### Mutation Spectra

To determine the mutation spectra of diploid mutator strains, we used plasmid shuffling to introduce the *pol3* alleles into BP9101, selecting transformants on SC plates lacking uracil and leucine. We wanted to score the mutation spectra after 17 to 20 cellular generations to allow sufficient numbers of mutations to accumulate. Since *pol3*-*01,L612M* is lethal as the sole source of Pol δ in these strains, we maintained *POL3/pol3*-*01,L612M* cells in the heterozygous state by serially diluting transformants on SC plates lacking uracil and leucine to get well-isolated, independent colonies. For all other alleles, we first serially diluted the colonies on FOA media to isolate single colonies in which the LEU2-CEN plasmid was the sole source of Pol δ. We then subcloned these colonies on FOA media. Colonies selected for whole genome sequencing were grown overnight in 3 ml YPD, and genomic DNA was purified from 10^8^ cells using a ZR Fungal/Bacterial Miniprep kit (Zymo Research). DNA was simultaneously fragmented and ligated to Illumina DNA adapters using the Nextera V2 Kit (Illumina), post-indexed by PCR, and sequenced using 101 bp, paired-end reads on an Illumina 2500 platform.

Reads were aligned to the *S. cerevisiae* S288C genome (Assembly R64-1-1) using the Burrows-Wheeler Aligner (Li-a). The aligned reads were then filtered to remove unmapped reads, non-uniquely mapping reads, and PCR duplicates using Picard and Samtools using standard settings (Li-b, URL: http://picard.sourceforge.net). After processing, the final average sequencing depth ranged between 100 and 300-fold. Coding variants were identified using VarScan2 with the strand bias filter option invoked (Kobolt). Only regions of the genome with >20-fold coverage that were not the site of a strain-specific SNP were evaluated^32^. Genomic positions where VarScan2 indicated a variant occurred between 40 and 60 percent of the reads mapping to that position were manually verified as heterozygous. Those that fell outside this range or occurred in low complexity regions, as determined by RepeatMasker, were not scored.

We assessed mutation frequencies by dividing the number of each class of mutations by the number of scored nucleotides in the genome that could produce that class of mutation (e.g. C → T/G → A mutations divided by all G or C bases). For frameshifts and larger insertion/deletion (indel) mutations we divided the number of mutations by the total number of bases sequenced. We determined the fraction of each mutation class by dividing the number of observations by the total mutations observed.

## Additional Information

**How to cite this article:** Dennis, D. G. *et al*. Normally lethal amino acid substitutions suppress an ultramutator DNA Polymerase δ variant. *Sci. Rep.* **7**, 46535; doi: 10.1038/srep46535 (2017).

**Publisher's note:** Springer Nature remains neutral with regard to jurisdictional claims in published maps and institutional affiliations.

## Supplementary Material

Supplementary Information

## Figures and Tables

**Table 1:** Influence of *eex* alleles on mutation rates.

		*MSH2/MSH2*^b^		Significant *p* values^c^	*eex* effect^d^	*msh2Δ/msh2Δ*^e^		Significant *p* values^c^	*eex e*ffect^d^
Allele		MR	CI			MR	CI		
*POL3*		2.2	(1.3, 3.4)			70	(45, 99)		
+	*S319F*	7.4	(4.8, 11)	0.001	3.3	490	(390, 600)	<0.0001	7.1
+	*G447D*	1.8	(0.9, 3.1)	ns	0.8	76	(59, 94)	ns	1.1
+	*F467I*	6.5	(3.9, 10)	0.018	2.9	85	(62, 110)	ns	1.2
+	*K559N*	8.5	(4.3, 15)	0.014	3.8	—			
+	*S611Y*	2.8	(0.9, 6.5)	ns	1.3	74	(57, 92)	ns	1.1
+	*R658G*	5.5	(3.0, 9.2)	ns	2.5	120	(99, 150)	0.048	1.8
+	*R674G*	—				—			
+	*Q697R*	—				—			
+	*A704V*	4.9	(1.9, 9.8)	ns	2.2	160	(120, 190)	0.0017	2.2
+	*G818C*	7.2	(2.9, 15)	ns	3.2	870	(600, 1200)	<0.0001	12.5
*pol3*-*01*		180	(140, 220)			11000	(9100, 13000)		
+	*S319F*	6.8	(4.5, 9.7)	<0.0001	0.04	1900	(1600, 2300)	<0.0001	0.17
+	*G447D*	15	(11, 19)	<0.0001	0.08	6200	(4900, 7600)	0.0007	0.56
+	*F467I*	9.3	(6.5, 13)	<0.0001	0.05	2600	(2100, 3100)	<0.0001	0.23
+	*K559N*	20	(10, 35)	<0.0001	0.11	—			
+	*S611Y*	31	(21, 43)	<0.0001	0.17	5200	(4100, 6400)	<0.0001	0.47
+	*R658G*	40	(29, 52)	<0.0001	0.22	7200	(5800, 8800)	0.017	0.65
+	*R674G*	—				—			
+	*Q697R*	—				—			
+	*A704V*	7.3	(4.4, 11)	<0.0001	0.04	1700	(1400, 2100)	<0.0001	0.16
+	*G818C*	7.7	(3.1, 16)	<0.0001	0.04	2400	(1800, 3200)	<0.0001	0.22
*pol3*-*L612M*		13	(9, 17)			2700	(2000, 3600)		
+	*S319F*	5.5	(3.6, 7.9)	0.012	0.44	1200	(990, 1500)	0.0004	0.45
+	*G447D*	4.6	(3.0, 6.5)	0.0006	0.37	1700	(1400, 2000)	ns	0.63
+	*F467I*	7.0	(4.5, 10)	ns	0.56	160	(120, 210)	<0.0001	0.06
+	*K559N*	4.1	(2.4, 6.6)	0.001	0.33	260	(210, 310)	<0.0001	0.10
+	*S611Y*	5.1	(3.2, 7.7)	0.008	0.41	350	(260, 460)	<0.0001	0.13
+	*R658G*	6.9	(3.8, 11)	ns	0.55	720	(600, 840)	<0.0001	0.26
+	*R674G*	2.7	(1.1, 5.1)	0.0001	0.21	370	(300, 440)	<0.0001	0.13
+	*Q697R*	2.0	(0.8, 3.8)	<0.0001	0.16	490	(360, 630)	<0.0001	0.18
+	*A704V*	3.1	(2.0, 4.4)	<0.0001	0.24	230	(190, 280)	<0.0001	0.08
+	*G818C*	3.6	(2.0, 5.7)	0.0001	0.28	810	(630, 1000)	<0.0001	0.29
*pol3*-*01,L612M*^f^		—				—			
+	*S319F*	640	(450, 860)			18000	(15000, 21000)		
+	*G447D*	8000	(55000, 11000)			13000	(10000, 16000)		
+	*F467I*	11000	(8200, 13000)			21000	(18000, 25000)		
+	*K559N*	2.1	(0.7, 4.9)			3200	(2600, 3800)		
+	*S611Y*	250	(200, 310)			6400	(5300, 7600)		
+	*R658G*	8700	(6900, 11000)			55000	(41000, 70000)		
+	*R674G*	4.6	(2.0, 8.8)			3900	(2600, 5400)		
+	*Q697R*	17	(4.2, 44)			4400	(3500, 5200)		
+	*A704V*	38	(20, 65)			5100	(4000, 6400)		
+	*G818C*	8.8	(1.5, 27)			4600	(3300, 5900)		

^a^Rates of canavanine-resistant (Can^r^) mutants per cell division (x 10^−7^) were determined by fluctuation analyses from multiple independent strain isolates using the R statistical package, rSalvador. Confidence intervals (95%) are in parentheses. A dash (—) indicates inviability.

^b^The strain was BP8001.

^c^Significant differences between the mutation rates conferred by *POL3, pol3*-*01*, or *pol3*-*L612M* and their respective *pol3*-*eex, pol3*-*01,eex*, or *pol3*-*L612M,eex* alleles were determined using the Likelihood Ratio Test in rSalvador and corrected for multiple testing using the Bonferroni method in p.adjust, found in the basic R statistical package. *p*-values greater than 0.05 were designated as not significant (*ns*).

^d^The *eex* effect was determined by dividing the mutation rate in *pol3*-*eex, pol3*-*01,eex*, or *pol3*-*L612M,eex* mutant cells by the mutation rate observed in control cells expressing *POL3, pol3*-*01* or *pol3*-*L612M*.

^e^The strain was BP9101.

^f^The mutation rates of all *pol3*-*01,L612M,eex* strains are as reported in Herr *et al*., Genetics, 2014.
