# Supplementary material for: Normally lethal amino acid substitutions suppress an ultramutator DNA Polymerase δ variant
Source: Sci Rep. 2017 Apr 18;7:46535. doi: 10.1038/srep46535 (PMC5394481; doi:10.1038/srep46535)
Supplement: Supplementary Information [file srep46535-s1.pdf]

Normally lethal amino acid substitutions suppress an ultramutator DNA Polymerase δ variant.

Daniel G. Dennis, Jill McKay-Fleisch, Kaila Eitzen, Ian Dowsett, Scott R. Kennedy, and Alan J. Herr*

Supplementary Information

Figure S1. B-family polymerase alignment. Aligned sequences of four B-family DNA polymerases are shown: bacteriophage T4 (T4 Pol), *Thermococcus gorgonarius* (T.g. pol B), *Saccharomyces cerevisiae* (S.c. pol d), and *Homo sapiens* Pol δ (H.s. pol d). Secondary structural elements of yeast Pol δ[49] are indicated below the alignment and color coded to depict their locations in the amino (gray), exo (red), palm (purple), fingers (blue) and thumb (green) domains of the protein: rectangles, α-helices; arrows, β-strands; solid lines, loops; dashed lines, structure not solved. Highlighted amino-acid residues in the alignment correspond to the following: red, absolutely conserved; yellow, identical in majority of sequences; gray, similar in majority of sequences. Conserved polymerase and exonuclease motifs are indicated by colored frames: blue, exo motifs; green, Pol motifs[80]; brown, motifs A, B, C[81]. Amino-acid substitutions of interest are placed above or below the alignment at the relevant position and highlighted according to the following scheme: red, *pol3-01,L612M* suppressors; yellow, *pol3-01* or *pol3-L612M*; no highlight, antimutators isolated as suppressors of the *pol3-01*, *pol3-D407A*, *pol3-F406A*, or *pol3-D463A*[39] proofreading-deficient alleles; gray, previously reported antimutators in Pol δ (*pol3-t* (D643N), G447S, and V758M)[51,70,82].

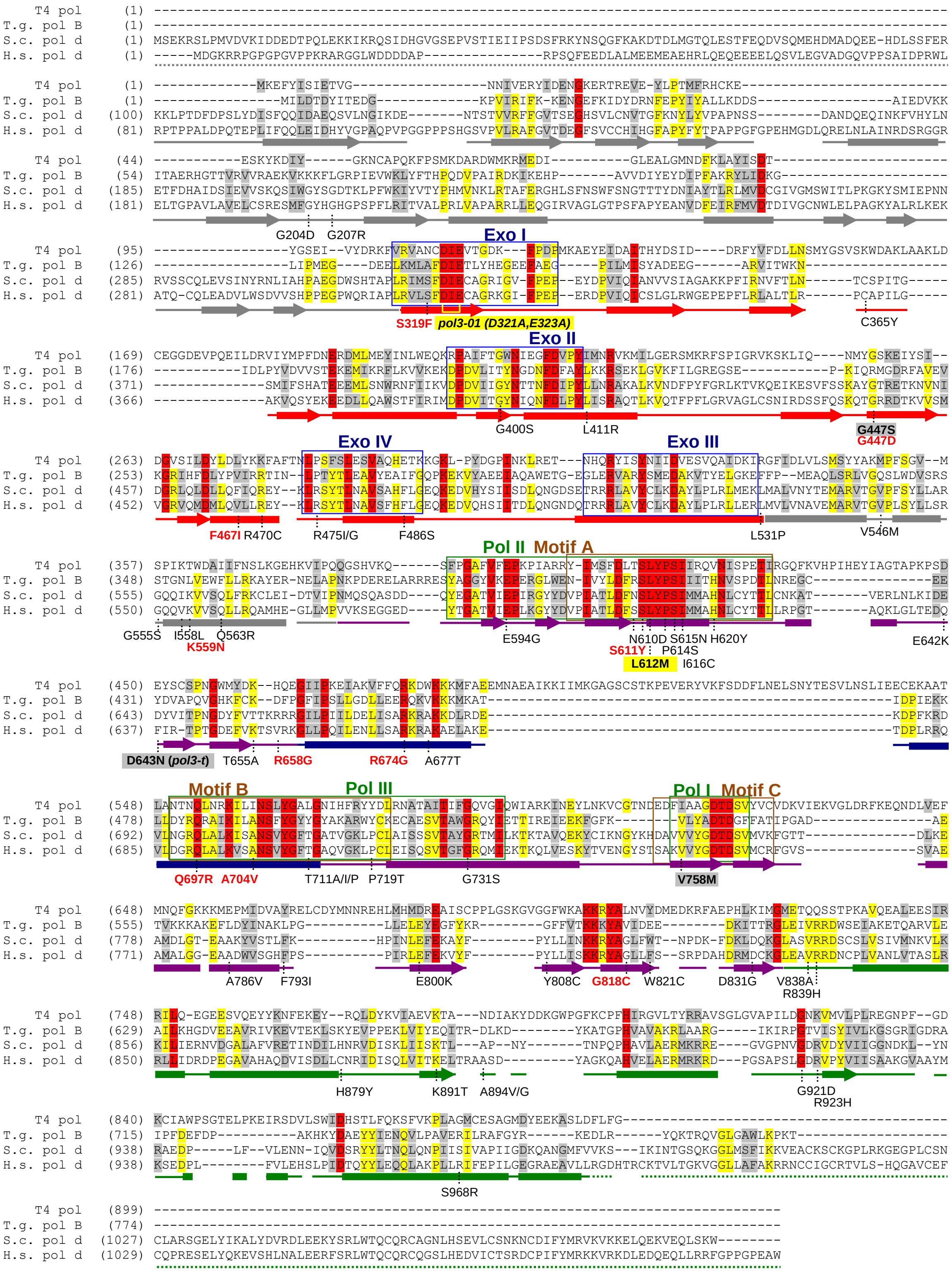

T4 pol
T.g. pol B
S.c. pol d
H.s. pol d
Exo I
Exo II
Exo III
Exo IV
Pol II
Motif A
Motif B
Pol III
Pol I
Motif C
G204D G207R
S319F pol3-01 (D321A,E323A)
C365Y
G400S L411R
G447S
G447D
F467I R470C R475I/G F486S L531P V546M
G555S I558L Q563R
K559N
E594G
N610D S615N H620Y
S611Y P614S
L612M I616C
E642K
D643N (pol3-t) T655A R658G R674G A677T
Q697R A704V T711A/I/P P719T G731S V758M
A786V F793I E800K Y808C G818C W821C D831G V838A R839H
H879Y K891T A894V/G G921D R923H
S968R
